# Neighborhood consistency in mental arithmetic: Behavioral and ERP evidence

**DOI:** 10.1186/1744-9081-3-66

**Published:** 2007-12-28

**Authors:** Frank Domahs, Ulrike Domahs, Matthias Schlesewsky, Elie Ratinckx, Tom Verguts, Klaus Willmes, Hans-Christoph Nuerk

**Affiliations:** 1 Interdisziplinäres Zentrum für Klinische Forschung BIOMAT., Universitätsklinikum der RWTH Aachen, Germany; 2 Lehr- und Forschungsgebiet Neuropsychologie, Universitätsklinikum der RWTH Aachen, Germany; 3 Institut für Germanistische Sprachwissenschaft, Philipps-Universität Marburg, Germany; 4 Department of Experimental Psychology, Ghent University, Belgium; 5 Fachbereich Psychologie, Paris Lodron Universität Salzburg, Austria

## Abstract

**Background:**

Recent cognitive and computational models (e.g. the Interacting Neighbors Model) state that in simple multiplication decade and unit digits of the candidate answers (including the correct result) are represented separately. Thus, these models challenge holistic views of number representation as well as traditional accounts of the classical problem size effect in simple arithmetic (i.e. the finding that large problems are answered slower and less accurate than small problems). Empirical data supporting this view are still scarce.

**Methods:**

Data of 24 participants who performed a multiplication verification task with Arabic digits (e.g. 8 × 4 = 36 - true or false?) are reported. Behavioral (i.e. RT and errors) and EEG (i.e. ERP) measures were recorded in parallel.

**Results:**

We provide evidence for neighborhood-consistency effects in the verification of simple multiplication problems (e.g. 8 × 4). Behaviorally, we find that decade-consistent lures, which share their decade digit with the correct result (e.g. **3**6), are harder to reject than matched inconsistent lures, which differ in both digits from the correct result (e.g. 28). This neighborhood consistency effect in product verification is similar to recent observations in the production of multiplication results. With respect to event-related potentials we find significant differences for consistent compared to inconsistent lures in the N400 (increased negativity) and Late Positive Component (reduced positivity). In this respect consistency effects in our paradigm resemble lexico-semantic effects earlier found in simple arithmetic and in orthographic input processing.

**Conclusion:**

Our data suggest that neighborhood consistency effects in simple multiplication stem at least partly from central (lexico-semantic') stages of processing. These results are compatible with current models on the representation of simple multiplication facts – in particular with the Interacting Neighbors Model – and with the notion of decomposed representations of two-digit numbers in general.

## Background

### Representation, production, and verification of multiplication facts

There is broad agreement that most simple multiplication facts are stored in and retrieved from an associative network in declarative memory [1-5]; for an overview see [6]. The structure of their representation in declarative memory is reflected by characteristic effects in processing. For instance, decreased response times (RTs) and error rates are robustly found for small compared to large problems [7-10] and for problems of the five table compared to non-five problems [11,12]. In addition to semantic features characterizing a specific *problem*, production of an incorrect *answer *also depends on the semantic relationship between this incorrect answer and the correct result. First, the majority of all multiplication errors can be classified as operand-related', i.e. they are multiples of one of the operands (e.g. 8 × 4 = 28) [10,11,13]. Furthermore, errors are more likely if they are numerically close to the correct result [4,13]. A similar effect is found in verification tasks: Operand-related incorrect probes (lures'), which are numerically close to the correct result, are harder to reject than neutral' lures [14].

Although all of these semantic relations may influence the retrieval and verification of multiplication facts, it seems plausible that different semantic features are available at different stages of processing. In particular, one may distinguish features that are related to properties of the *problem *(e.g. the operand rows involved or the size and parity status of the operands) and features that are related to the correct *result *(e.g. its relatedness to other answer candidates). Features related to the problem should be available at an earlier processing stage than features related to the correct result. For instance, the problem 9 × 8 involves the multiplication rows of 9 and 8. It contains two relatively large operands, one of them being odd and the other one being even. These features are related to the problem itself and not to the result. On the other hand, although 18, 27, 36, 64, and 81 are all candidate answers which are semantically related to the correct result 72, only the latter two can be regarded as immediate neighbors of the correct answer. To know this, one has to have at least partial knowledge of the magnitude of the correct result. Problem-related features as parity or the multiplication rows involved may even influence the choice of an appropriate solution strategy [15]. In production paradigms, this is obviously not possible for result-related features. In verification tasks, more global semantic properties of the probe (e.g. familiarity as multiplication result [16] or the parity pattern of operands and probe) may be used earlier than specific semantic properties related to the correct result (e.g. split between a lure and the correct result). Information which is available early in processing may still influence strategy choice which is not possible for information which is available only late.

To give an example, a verification study by Lemaire and Fayol [17] examined the influence of parity knowledge on product verification. In multiplication, only two odd operands lead to an odd result while at least one even operand suffices to lead to an even result. This more global, problem- or operation-related type of knowledge can be used to quickly reject lures if they violate the parity rule (see also [16,18]). Crucially, Lemaire and Fayol [17] found that a strategy based on parity judgments was not used to the same extent in all conditions. Rather, its use increased with increasing problem difficulty and with decreasing Stimulus Onset Asynchrony (SOA) between stimulus (problem) and probe. Both findings suggest that there is a race between the actual retrieval of the correct result from memory and plausibility strategies in product verification. With easy problems and/or sufficient time available, direct retrieval may be fast enough, while with difficult problems and/or little time plausibility judgments may come into play. Thus, the use of different SOAs between stimulus and probe may help to uncover the point in time at which different kinds of semantic knowledge are available.

Semantic knowledge accessible at approximately the same stage of processing may not lead to additive effects in product verification. Indeed, Masse and Lemaire [19] observed that their participants did not combine the parity rule and the five rule (if one operand = 5, the result must end in 0 or 5) to verify multiplication problems: Although the parity as well as the five rule proved to be effective, there was no additional gain, if a lure violated both rules at the same time. Note that both rules are problem-related, representing approximately the same level of semantic specificity. However, the pattern is different if semantic knowledge with different degrees of specificity (i.e. more general vs. more specific knowledge) is involved. For instance, the effects of operand-relatedness and numerical distance (split) of a given lure from the correct answer have been shown to be additive [9,20]. It can be assumed that the more global property of operand-relatedness comes into play at an earlier stage of processing compared to a more specific relationship of a lure with the correct result like their numerical distance.

### EEG correlates of simple multiplication

Only few electrophysiological studies have addressed representation and retrieval of arithmetic facts. Jost, Niedeggen, and Rösler were the first to apply the method of event-related potentials to simple multiplication problems [20-23]. In product verification, these authors found components similar to those known from the domain of verbal lexico-semantic processing, which they referred to as arithmetic N400 and Late Positive Component (LPC). In both components, activation was inversely proportional to the semantic relatedness of the probe with the correct result [20]. Specifically, the N400 and LPC amplitudes were more pronounced for unrelated (e.g. 5 × 8 = 34) compared to operand-related lures (e.g. 8 × 4 = 24) and for numerically distant compared to close lures. As for the behavioral results, the effects of operand-relatedness and split of the lure to the correct result proved to be additive in the LPC. However, this was not the case for the N400, where numerical distance influenced the amplitude of related but not of unrelated lures. N400 activation is assumed to reflect the amount of activation spread that originates from a prime (i.e. the given problem) to the target (i.e. the lure). The LPC, an electrophysiological response possibly originating from the family of P300 effects, is interpreted in terms of plausibility evaluation or detection of unexpected events (surprise'), still uncontaminated by response-preparation processes. Note that in this account, both the N400 and the LPC effect are explained in terms of semantic relatedness, irrespective of any formal overlap as, for example, phonological or orthographic similarity. This kind of semantic effect, which is not dependent on formal overlap, will be referred to as semantic' hereafter.

### Neighborhood consistency in simple multiplication

In addition to the features reviewed above, a recent model on multiplication fact retrieval, proposed by Verguts and Fias [5], predicts that another feature – neighborhood consistency – is involved in production and verification of simple multiplication results. According to this *interacting neighbors *(IN) model, problem operands (e.g., 7 and 4) activate nodes in a semantic field, each of which responds most strongly to a particular operand pair: For example, the semantic field node (7, 4) responds most strongly to problem 7 × 4, more weakly to 7 × 3, and hardly at all to 9 × 2. Semantic field nodes in their turn activate nodes in a subsequent decade field: Thus, the semantic field node in our example activates the 2-node in the decade field because 7 × 4 equals 28. In parallel, semantic field nodes also activate nodes in a unit field: For example, 7 × 4 activates the 8-node there because 8 is the unit of the correct solution. At this processing stage, there is inhibition and/or facilitation between decade and unit digits of all activated answer candidates. As a result, the IN model predicts that in production correct results sharing a digit with many competing incorrect answers (neighbors') are facilitated compared to correct results sharing their digits with only few or no neighbors at all. For example, the result 28 of the problem 7 × 4 shares its decade digit with the results **2**0, **2**1, and **2**4 associated with neighboring problems 5 × 4, 7 × 3, and 6 × 4, and because the respective semantic field nodes (5, 4), (7, 3), and (6, 4) are (weakly) activated by the problem 7 × 4, they also partly activate the 2-node in the decades field. On the other hand, no such facilitation from neighboring problems arises from the neighbors of 72, the correct result of the problem 8 × 9 because none of its neighbors results in a decade 7 response. Neighbors sharing a digit are called consistent', other neighbors inconsistent'. The number of consistent neighbors systematically decreases with increasing problem size, and it has been argued that in the light of this relationship neighborhood consistency may explain the classical problem size effect, i.e. the fact that large problems lead to slower responses and more errors than small problems [5,24,25]. Furthermore, according to the IN model neighboring errors which share a digit with the correct result (consistent errors'; e.g., **2**0, **2**1, and **2**4 for problem 7 × 4) should be more likely to be produced than inconsistent errors. Recently, Domahs and colleagues [24] provided evidence for such consistency effects in the verbal production of multiplication facts.

### The present study

The present investigation aims at providing empirical evidence for the consistency effect in simple multiplication and to elucidate its functional locus. It will do so in a combined behavioral and ERP paradigm. Behaviorally, we will try to establish the consistency effect in a task which does not include any overt verbal response – product verification. Furthermore, the consistency effect will be compared with the well-known relatedness effect in simple multiplication to uncover the sequence of semantic activation involved. With respect to the electrophysiological part of the study, we will examine ERP correlates of the consistency effect. Crucially, the specific nature of ERP components should provide an answer to the question whether the consistency effect is based on central (lexical or semantic) versus purely peripheral processing, the latter comprising stimulus perception, answer selection and production. Both lines of arguments will be explained in more detail in the following section.

At the behavioral level, Domahs and colleagues [24] have demonstrated in a production paradigm that units and decades of multiplication facts are separately processed at some stage, but this does not imply that units and decades are *represented *separately. For instance, they could be holistically represented in lexico-semantic memory, but only processed separately at a peripheral production stage, and there lead to a consistency effect. To test this, one needs to investigate whether the consistency effect can also be observed in tasks without any verbal production. If the hypothesis of an involvement of lexico-semantic representations in the consistency effect is correct, then a consistency effect should also be observed in a task without any verbal output. In the present study, we therefore investigate whether the neighborhood consistency effect also holds for the verification of simple multiplication facts, where the subjects are asked to give a dichotomous manual response, but no verbal response is required. Hence, we investigate whether decade-consistent lures (e.g. 8 × 4 = **3**6 or **3**7) are harder to reject than otherwise comparable inconsistent lures (e.g. 8 × 4 = 28 or 29).

Interestingly, an effect similar to the consistency effect is observed in a formally similar psycholinguistic task – lexical decision. In visual lexical decision (i.e. the speeded classification of a letter string as word or pseudoword) the existence of many neighbors' (i.e. words which share all but one letter with the target) slows down the answer to pseudowords (neighborhood size effect; [26,27]). Crucially, the existence of highly frequent neighbors makes it particularly hard to reject a pseudoword (neighborhood frequency effect; [26-28]). To see the similarity between the latter effect and the consistency effect in product verification, consider why there is a neighborhood frequency effect in lexical decision according to the interactive activation model of word recognition [29]: Rejecting a pseudoword like *kand *may be hindered by a competing word like *sand *if this word is formally similar (i.e. a neighbor) and of high frequency (since high frequency leads to high baseline activation). However, a pseudoword like *kulf *would not suffer from significant interference from a single infrequent neighbor like *gulf*. Without any formal overlap between the pseudoword tested and existing words no interference is assumed to occur at all. In a multiplication verification paradigm, one might consider the correct multiplication result to be a high frequent neighbor of a consistent lure. When a consistent lure is presented, the correct result is partially activated for two reasons: First, it receives activation from the problem. Second, it is formally related to (i.e. shares one digit with) the lure. Accordingly, a lure which has formal overlap with the correct result (i.e. a consistent lure) may be harder to reject than a lure without such formal overlap with this familiar neighbor (i.e. an inconsistent lure). For instance, given the problem 8 × 4, the lure **3**6 shares all but one digit with the correct result **3**2, which is not true for the lure 28. In this sense, the explanation given by the interactive activation model for the neighborhood frequency effect and the explanation given by the IN model for the neighborhood consistency effect are conceptually similar.

Although neighborhood effects in lexical decision may be explained in terms of semantic activation of the neighbors [30], it should be pointed out that they are necessarily based on formal overlap of orthographic lexical entries. Thus, they differ from other semantic effects, which do not depend on formal overlap (see above). Therefore, we will use the term lexical' for effects which require formal overlap, even though they may also be semantic in nature, in order to distinguish them from purely semantic' effects (e.g. effects of semantic category).

The expected consistency effect will be compared to the well established relatedness effect, i.e. the finding that operand-related lures (e.g. 8 × 4 = 28 or 36) are harder to reject than unrelated lures (e.g. 8 × 4 = 29 or 37). Given that consistency is defined in terms of digit identity with the correct result, it requires specific, result-related semantic knowledge. If the correct result itself is not sufficiently activated, any relation (like consistency) to the correct result can hardly be sufficiently activated. This is different for the relatedness effect. It is well conceivable that initially all entries of both multiplication rows involved are activated to a similar extent, before the activation of the correct result raises. Thus, in contrast to the consistency effect, no knowledge of the actual result is necessary for the activation of operand-related candidates. Therefore, we predict that the consistency effect should appear later than the relatedness effect. This issue will be tackled using two different SOAs between presentation of the problem and the lure, adopting the method described by Lemaire and Fayol [17]. In addition to a trivial main effect of SOA (probes after a long SOA should be responded to faster than probes after a short SOA) we expect an interaction between consistency, relatedness and SOA: While relatedness effects should already be apparent after a short SOA as unrelated probes can be rejected based on familiarity, consistency effects may only be observed after a longer SOA, i.e. after the correct result has received sufficient activation.

As stated above, the aim of the electrophysiological part of the present study is to resolve the question whether the consistency effect is only related to peripheral (i.e. encoding and/or production) stages or also to central lexico-semantic' processing stages. To this end, we will follow the argumentation of different researchers [26,30,31] for the processing of verbal orthographic input and of Jost, Niedeggen, and Rösler [20-23] for the verification of arithmetic problems. In line with these authors, we assume that if the consistency effect – similar to the relatedness effect – has its origin at lexico-semantic processing stages, we should find significant differences in EEG components known to reflect this stages of processing, i.e. the N400 and the LPC. In contrast, if the consistency effect is purely peripheral, we will observe no N400 and LPC effects for consistency. Note, however, that we only predict the *occurrence *of systematic N400 and LPC effects related to decade-consistency (if this effect originates at a lexico-semantic stage of processing), but not their specific *direction*. In this respect, a semantic' account, solely based on semantic relatedness, leads to different expectations than a lexical' account based on neighborhood characteristics and thus also on formal overlap. In fact, if the consistency effect is due to purely semantic interference without formal overlap (as is the case for the relatedness effect), then we should observe a more pronounced N400 for inconsistent compared to consistent lures, because the former are semantically less related to the correct result than the latter (for relatedness effects in the N400 component see [20,22,23]). Thus, if this semantic account' holds, consistency and relatedness effects should have the same direction. On the other hand, consistent lures have a familiar neighbor (i.e. the correct result, sharing their decade digit) and inconsistent lures have no such familiar neighbor. In analogy to visual lexical decision [26,30,31], then, we would expect a more pronounced N400 effect for consistent answers, which do show formal overlap, compared to inconsistent answers, which don't have formal overlap with the correct result (lexical' account) – i.e. the opposite of what is expected based on semantic relatedness. The same argument holds for the LPC where the expectation also differs depending on whether formal overlap is involved or not. Importantly, both the lexical and the semantic account are based on central (i.e. lexico-semantic) rather than peripheral (e.g. encoding) stages of processing, which is the main distinction to be addressed in the present study.

In sum, in the present investigation we try to pinpoint the functional locus of the consistency effect in simple multiplication using a combination of behavioral and electrophysiological methods. If the predictions of the Interacting Neighbors model hold, we expect to find evidence for consistency effects. Specifically, we expect longer RTs and higher error rates when consistent probes compared to inconsistent probes have to be rejected. Given that more specific knowledge of the correct result is necessary, consistency effects should occur later than the relatedness effect, i.e. after a longer SOA. Finally, if consistency effects at least partly stem from central lexico-semantic' stages of representation, we expect significant differences between consistent and inconsistent trials in specific ERP components (N400 and LPC).

## Methods

### Participants

Forty-four right handed students of the University of Marburg took part in the experiment. All gave their informed consent. They were naïve with respect to the specific purpose of the experiment. For their participation they received a compensation of € 15. Twenty participants had to be excluded from the analyses (see the Analyses section below) due to high error rates or eye movement artifacts. The mean age of the remaining 24 participants (11 women) was 23 years (range from 19 to 29 years).

### Stimuli

All 18 experimental stimulus sets are listed in Additional file 1. Each stimulus set consisted of a multiplication problem with its correct answer and four incorrect probes (lures'). Only problems with one-digit operands were presented. Operands 0 and 1 were not used in order to avoid rule-based processes [21]. Half of the problems were followed by a lure, the other half by a correct probe. Therefore, the correct probe was presented four times per stimulus set, each lure only once. All correct probes and lures were two-digit numbers consisting of different digits.

Lures were manipulated in a 2 (relatedness) × 2 (decade-consistency) design. Therefore, half of the probes were operand-related (e.g. 8 × 4 = 28 or 36) and the other half unrelated, i.e. not belonging to any conventional multiplication table (e.g. 8 × 4 = 29 or 37). Furthermore, half of the lures were decade-consistent, i.e. containing the same decade digit as the correct result (e.g. 8 × 4 = **3**6 or **3**7) and the other half decade-inconsistent (e.g. 8 × 4 = 28 or 29).

For both factors (relatedness and consistency) the distance of the lures from the correct result (split) was matched (mean split for related consistent: 5.4; related inconsistent: 5.4; unrelated consistent: 5.1; unrelated inconsistent: 5.1). Note that if the very small difference in mean split between related and unrelated probes has any consequence at all, then it should work against the expected relatedness effect: Lures with a large split are typically easier to reject than probes with a small split [2,14] while related probes should be harder to reject than unrelated probes.

Parity of consistent and inconsistent probes was matched, because both members of a pair of probes – deviating by plus or minus one operand respectively – have the same parity (e.g. result: 7 × 6, probes: 47 and 31; Although both probes are odd and thus deviate from the parity status of the correct result [42 = even], they do so in the same way.). Parity deviated from the correct result in 11 pairs of operand-related probes and in 10 pairs of unrelated probes (see Additional file 1).

Furthermore, lures did not repeat operands in a congruent position (e.g. 8 × **4 **= 2**4 **or **2 **× 7 = **2**1) to avoid operand intrusion based interference [4,13]. However, stimuli were included when the first operand of the problem reappeared in an *in*congruent position – i.e. as unit digit – of the probe (e.g. **7 **× 3 = 1**7**). In such items, only very weak operand interference may be expected: First, intrusion of an operand into an incongruent position of the result leads to much less interference than intrusion into a congruent position [4]. Moreover, intrusion errors related to the first operand are much less frequent than intrusion errors related to the second operand [4,13,32]. Finally, for unrelated probes the distribution of possible intrusion effects even worked against our hypotheses. In this condition, five inconsistent and four consistent probes were affected by potential incongruent intrusion (see Additional file 1). While we hypothesized that inconsistent probes are easier to reject, incongruent intrusions should – if anything – make the decision more difficult.

Moreover, operand-related lures were equally often related to the first or to the second operand and always deviated from the correct result by exactly one step in the multiplication row. The same lure was only used for a problem in one operand order and not for the complementary operand order (e.g. 24 was used for 3 × 6, but not for 6 × 3). If a given lure smaller than the correct result contained a 5 or 0 in the unit position, the same was true for its counterpart larger than the correct result (e.g. 7 × 5 = 30 or 40), to avoid a confounding influence of the five effect.

Each experimental stimulus set was presented twice (once per SOA) with each of the four lures and eight times (four times per SOA) with the correct probe. Altogether, each participant's data set consisted of 36 items per lure type and 144 correct probes. To balance the frequency of occurrence of a specific number as a correct probe or a related lure, 44 filler items were introduced. The same balancing procedure was not possible for unrelated lures, as these probes cannot – by definition – be a correct answer of a multiplication problem. Problems were presented in pseudo-randomized order such that no operand appeared in the same position in a sequence. The same problem (including the complementary operand order) could only be repeated after at least four different intervening problems. No more than four consecutive correct probes or lures occurred.

### Procedure

Participants had to judge whether the proposed solution of a simple multiplication problem (probe) was correct or not. First, they received written instructions about the task, after which they could clarify any remaining doubts with the experimenter. Instructions emphasized the aim to respond as quickly and accurately as possible. Participants sat in an armchair in a dimly illuminated and electrically shielded room, facing a computer monitor placed 150 cm in front of them. All stimuli were presented in Times New Roman font type and NRC7BIT 172 size (approximately 2.0 cm height and up to 1.4 cm width per digit) in central position on the screen using ERTS 3.32 software (BeriSoft Cooperation, Frankfurt, Germany). Participants were instructed to maintain visual gaze on the central fixation point and to restrain from blinking during active task periods.

Each trial began with an ×', presented for 300 ms and followed by a blank screen, which lasted for 200 ms. Then, both operands were presented simultaneously in Arabic digits for 100 ms, separated by two blank spaces. No multiplication sign was presented between the operands to speed up perception of the problem (cf. [33]). After presentation of the problem, a blank screen appeared for 50 ms or 450 ms (short or long SOA conditions, respectively). Finally, the probe was presented in Arabic digits until response or until 2000 ms were passed. There were two intertrial intervals: First, a ...' appeared, lasting for 1400 ms, to signal that participants could rest their eyes and blink, which was followed by a blank screen for another 100 ms. Thus, each trial lasted 4150 ms or 4550 ms (short or long SOA conditions, respectively). No feedback was provided.

Problems were presented in two blocks, each containing 288 experimental and 88 filler trials. In pseudo-randomized order, half of the trials had a short and the other half a long SOA. During one block the yes' response had to be given with the left hand and the no' response with the right hand, during the other block the response – hand assignment was reversed. The order of response-hand assignments was counterbalanced across participants. Responses of both blocks were collapsed for analyses. Each block was preceded by 20 training trails, which consisted exclusively of items of the filler set. After every 44 or 45 trials, a short break was provided. Both blocks together lasted approximately one hour.

### EEG-recording

The EEG was recorded by means of 22 AgAgCl electrodes via a *Brainvision *amplifier (Brain Products, Gilching, Germany) with the C2 electrode serving as ground electrode. The reference electrode during recording was placed at the left mastoid. EEGs were re-referenced off-line to both mastoids. To control for eye-movement artifacts, vertical eye movements were recorded by electrodes above and below the participant's left eye, and horizontal eye movements by two electrodes fixed to the outer canthus of both eyes. Electrode impedances were kept below 5 kΩ. EEGs and EOGs were recorded continuously with a digitization rate of 250 Hz, and filtered offline with a bandpass filter from 0.2 to 30 Hz.

### Analyses

Only RTs for correct responses were entered into the analyses. Furthermore, an outlier trimming procedure was applied which excluded all responses for which RT deviated by more than three standard deviations from the mean of the particular condition (relatedness × consistency × SOA). Altogether, 89 (2.58%) responses were identified as outliers.

ERPs were computed for each participant, condition, and electrode. Segments associated with erroneous responses were excluded as well as segments containing any EEG artifact > ± 50 μV in an epoch of -100 ms to +650 ms from the onset of probe presentation (21.8% of trials). To include an individual participant's data set into the analyses, a minimum of 15 appropriate EEG segments per condition was required. Following this procedure, the data of 24 out of the 44 participants could be included in the analyses. Apparently, some participants had problems to cope with task demands, leading to high error rates especially in the short SOA condition. Moreover, a substantial amount of eye blinking artifacts may be due to the instruction to answer as fast as possible, i.e. to the combination of EEG and RT measurements. Nevertheless, we preferred this combined approach as our interpretation is based on both sources of data (see Discussion).

In a repeated measurement design, the factors consistency (consistent vs. inconsistent lures) and relatedness (related vs. unrelated lures) were considered for each SOA (short or long) separately. For measurements of mean voltage, time windows were chosen according to SOA and component. Time windows were defined by visual inspection of grand average curves. For the short SOA condition, we chose time windows from 350 to 450 ms and from 450 to 650 ms post presentation onset of the probe. For the long SOA condition, we selected time windows from 300 to 400 ms and 475 to 600 ms. The ANOVAs were calculated in a 2 × 2 design (consistency × relatedness) for electrodes in particular regions, which were defined as the four quadrants of the scalp: left frontal (electrodes F3, F7, FC5), right frontal (F4, F8, FC6), left parietal (P3, P7, CP5), and right parietal (P4, P8, CP6). Main effects of the factor region will not be reported throughout this report for the sake of brevity.

## Results

### Behavioral data

Overall, correct probes were responded to faster than lures. However, as only lures are informative with respect to our hypotheses, we will only present analyses for these conditions. A 2 × 2 × 2 repeated measurements ANOVA using RT as dependent variable including the factors SOA (short vs. long), relatedness (related vs. unrelated lures), and consistency (consistent vs. inconsistent lures) revealed significant main effects for all three factors: Trials in the long SOA condition were responded to 174 ms faster than trials in the short SOA condition (F (1; 23) = 257.08; MSe = 5629.143; p < .001). Furthermore, related probes were rejected 100 ms slower than unrelated probes (F (1; 23) = 48.11; MSe = 9959.572; p < .001). Finally, consistent probes were rejected 23 ms more slowly compared to inconsistent probes (F (1; 23) = 21.58; MSe = 1146.464 p < .001).

In addition to the main effects, there was also an interaction between SOA and relatedness such that the relatedness effect was more pronounced in the short than in the long SOA condition (126 ms vs. 74 ms; F (1; 23) = 17.33; MSe = 1902.782; p < .001). Moreover, consistency also interacted with SOA (F (1; 23) = 5.35; MSe = 705.045; p < .05): The consistency effect was larger for the long SOA (30 ms) than for the short SOA (15 ms). Finally, there was a three-way interaction between SOA, relatedness, and consistency (F (1; 23) = 15.07; MSe = 500.198; p = .001), which was further explored by separate ANOVAs for the different SOAs (see Table 1).

**Table 1:** Mean reaction times and standard deviations in ms for correct responses.

**Condition**	**SOA**	
	
	**short**	**long**
**related consistent**	913 (210)	709 (184)
**related inconsistent**	879 (203)	683 (184)
**unrelated consistent**	767 (164)	640 (135)
**unrelated inconsistent**	773 (174)	604 (133)

Stimulus Onset Asynchrony (SOA) between onset of problem and answer presentation: short = 150 ms; long = 550 ms.

In the short SOA condition, there were again main effects of relatedness (F (1; 23) = 62.77; MSe = 6081.961; p < .01) and consistency (F (1; 23) = 4.87; MSe = 945.586; p < .05). Most importantly, we also obtained an interaction between relatedness and consistency (F (1; 23) = 9.69; MSe = 995.733; p < .01): While there was a consistency effect of 34 ms in the related conditions (t (23) = 3.23; p < .01), we found no such consistency effect (-6 ms) for unrelated trials (t (23) = 0.86; p = .40). However, in the long SOA condition the picture was different (see Table 1). While we also observed main effects of relatedness (F (1; 23) = 22.55; MSe = 5780.392; p < .001) and consistency (F (1; 23) = 26.41; MSe = 906.025; p < .001), we no longer observed an interaction between relatedness and consistency (F (1; 23) = 1.33; MSe = 453.159; p = .26). Thus, the three-way interaction in the overall ANOVA can be qualified as follows: Consistency and relatedness interacted for the short SOA, but not for the long.

On average, 9.75% errors were made. For the sake of brevity, the results are only shortly summarized. Overall, a very similar pattern was observed as for the RT data: Conditions, which were responded to more slowly, were also more error-prone. In the analysis of arcsine-transformed error rates, the same main effects for SOA, relatedness, and consistency reached significance. Additionally, the two-way interactions relatedness × SOA and consistency × SOA were significant, but – as in the RT data – in reverse directions: The relatedness effect was larger for the short SOA; the consistency effect was larger for the long SOA. Only the three-way interaction SOA × relatedness × consistency failed to reach significance for the error data.

### EEG-data

Overall, consistency and relatedness produced distinctive ERP effects in subsequent phases of cognitive processing. In the following, the electrophysiological effects are presented separately for SOA and time window.

#### Short SOA

**Figure 1:**
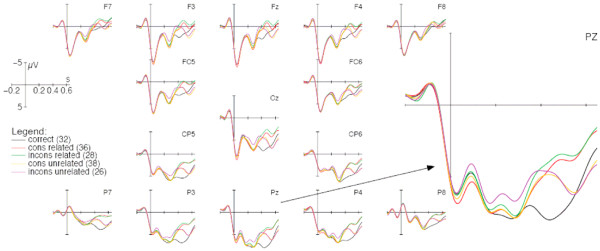
**Grand average ERPs with short (150 ms) SOA presentation**. On the right-hand side, the grand average ERP of electrode PZ is zoomed in for better readability. Stimulus onset of the probe is represented by the vertical microvolt calibration bar. Grand average curves are baseline-corrected in the time window 100 ms before stimulus onset. Note that negative voltages are plotted in the upward direction. Numbers in brackets are examples for probes presented after the problem 8 × 4. rel = related; unrel = unrelated; cons = decade-consistent; incons = inconsistent.

Figure 1 depicts grand averages for correct probes and for all four types of lures varying according to the factors consistency and relatedness. In general, the grand-average curves started with a positive-going deflection from the onset of segmentation up to 100 ms followed by a negativity from 100 to 200 ms and a positivity from 200 to 350 ms. In a time window from 350 to 450 ms post onset of probe presentation, each lure type elicited a negativity in comparison to the correct probe (F (1; 23) ≥ 17.807; MSe = 3.765; p < .001). A further analysis of the four lure types in a 2 × 2 × 4 (relatedness × consistency × region) repeated measurements ANOVA did not reveal any main effect (consistency: F (1; 23) < 1; MSe = 2.048; relatedness: F (1; 23) = 1.476; MSe = 1.595; p = .236) nor any interaction (consistency × relatedness: F (1; 23) < 1; MSe = 2.742; consistency × relatedness × region: F (3; 69) = 1.813; MSe = .366; p = .16). This picture changed in the subsequent time window from 450 to 650 ms, in which related probes separated from unrelated ones (F (1; 23) = 32.635; MSe = 7.461; p < .001). The factor consistency, however, did not play any role (F (1; 23) < 1; MSe = 3.482). As can be seen in Figure 1, grand average curves of unrelated conditions proceeded in a more positive direction compared to related conditions. This effect might indicate that unrelated answers could be rejected more easily than related answers or that they were judged as less plausible.

#### Long SOA

**Figure 2:**
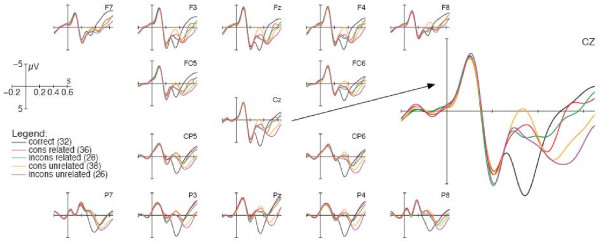
**Grand average ERPs with long (550 ms) SOA presentation**. On the right-hand side, the grand average ERP of electrode CZ is zoomed in for better readability. Stimulus onset of the probe is represented by the vertical microvolt calibration bar. Grand average curves are baseline-corrected in the time window 100 ms before stimulus onset. Note that negative voltages are plotted in the upward direction. Numbers in brackets are examples for probes presented after the problem 8 × 4. rel = related; unrel = unrelated; cons = decade-consistent; incons = inconsistent.

ERP curves obtained for conditions with a long SOA of 550 ms are depicted in Figure 2. In general, the first visible component for all the ERPs was a negative-going deflection occurring until about 150 ms post onset of the probe (N1). This was followed by a positive deflection occurring at approximately 200 ms (P2). Afterwards, the EEG evinced two components with differential curves for correct probes and lures (N400 and LPC), which will be presented in more detail in the order of their latency.

**Figure 3:**
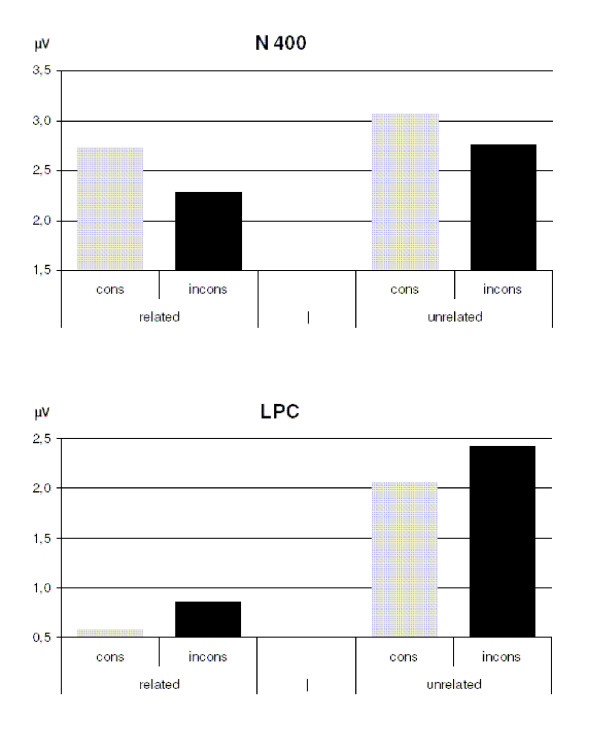
**Mean amplitudes of N400 and LPC components with long (550 ms) SOA presentation**. Plotted amplitudes are absolute differences to the correct condition. All difference values are based on the grand averages over all participants, over all twelve electrodes which entered into the analyses (see Methods section), and over the time windows 300 – 400 ms (N400) and 475 – 600 ms (LPC) post probe onset, respectively. cons = decade-consistent; incons = inconsistent.

In analogy to the findings for conditions with short SOA presentation, lures elicited a negative-going deflection compared to the correct condition in the time window between 300 and 400 ms post onset of the result (F (1; 23) ≥ 50.924; MSe ≤ 7.017; p < .001). However, in contrast to the results in the short SOA conditions, we also found qualitative differences between lure types (see Figure 3). A 2 × 2 × 4 (relatedness × consistency × region) repeated measurements ANOVA revealed main effects for the factor relatedness (F (1; 23) = 4.608; MSe = 3.718; p < .044) and for the factor consistency (F (1; 23) = 8.465; MSe = 1.58; p < .009). The negativity was more pronounced for unrelated than for related conditions, but also for consistent compared to inconsistent conditions (see Figures 2 and 3). Although the negativity effect seemed to be most pronounced in fronto-central regions, there was no interaction of the factor region with the factors relatedness (F (3; 69) < 1; MSe = .446) or consistency (F (3; 69) = 1,301: MSe = .757; p = .283) nor a three-way interaction (F (3; 69) < 1; MSe = .628).

With respect to the occurrence of a late positive component between 475 to 600 ms, not only unrelated conditions produced an enhanced positivity compared to related conditions (F (1; 23) = 52.704; MSe = 4.242; p < .001), but also inconsistent conditions compared to consistent lures (F (1; 23) = 4.767; MSe = 2.271; p < .04) (see Figures 2 and 3). An interaction was found for the factors relatedness × region (F (3; 69) = 6.91; MSe = .516; p < .002), but not for the factors consistency × region (F (3; 69) = 2.394; MSe = .52; p = .093), relatedness × consistency (F (1; 23) < 1; MSe = 3.666), or relatedness × consistency × region (F (3; 69) < 1; MSe = .483). A *post-hoc *analysis of the interaction relatedness × region revealed a significant relatedness effect in each quadrant (F (1; 23) ≥ 27.89; MSe ≤ 1.866; p < .001), the effect being most pronounced in parietal quadrants. Overall, the findings obtained for lures, which varied according to the factors relatedness and consistency, indicate that both factors influenced the mean voltage changes in the verification of arithmetic problems.

## Discussion

We reported data of a combined behavioral and ERP experiment. In the following section, we will first discuss behavioral data and afterwards EEG data. Finally, we will address the implications of our results for the functional localization of consistency effects in simple multiplication.

### Behavioral data

We demonstrated for the first time the consistency effect in a product verification task. Consistent neighbors were significantly harder to reject than inconsistent neighbors, while split, relatedness, and other known factors were controlled for. This is in line with the idea that multiplication facts are represented in a decomposed fashion such that – at a certain stage-decade and unit digits of the result are processed separately [4,5,24,25,34,35].

We also replicated the relatedness effect in multiplication: Related lures (e.g. 8 × 4 = 28 or 36), which are part of the multiplication table, were harder to reject than unrelated lures (e.g. 8 × 4 = 29 or 37), which are not included in the multiplication table. Furthermore, we observed the expected main effect of SOA: Participants responded significantly faster after a long than after a short SOA between problem and probe [17].

There were also instructive interactions between the above mentioned factors. On the one hand, the relatedness effect was larger in the short SOA condition. This interaction is consistent with the idea that non-table errors may be easily rejected – solely based on familiarity judgments – even when the correct answer has not yet been retrieved [16]. However, in the long SOA condition the correct response is already activated to a larger extent when the lure is presented and, consequently, familiarity with the lure may be less important to determine performance [17]. On the other hand, the interaction between consistency and SOA is the opposite of what we observed for the relatedness × SOA interaction: The consistency effect was larger (rather than smaller) in the long SOA condition. Different from the relatedness effect, which is based on familiarity with certain numbers as possible results within certain multiplication rows, the consistency effect relies on a specific relation between the correct result and its incorrect alternative. In the long SOA condition, the correct result is activated to a larger extent and therefore the specific relation (i.e. digit identity) between this correct result and the lure may determine performance. However, in the short SOA condition the correct result may not be fully activated yet. As the consistency effect is defined with respect to the relation between the not-yet fully processed correct result and the lure presented on the screen, the influence of consistency should be rather small. Thus, while some specific result-related knowledge is necessary for the consistency effect to appear, this is not true for the relatedness effect, which can already be observed at an earlier stage of processing. Finally, we observed a three-way interaction between all three factors (SOA, relatedness, and consistency). This was due to the fact that consistency and relatedness interacted in the short SOA condition but not in the long SOA condition. The above explanation is also in line with this pattern of results: After a long SOA, i.e. when the correct result is mostly available, both consistency and relatedness effects are observed in an additive fashion (without interaction). However, for the short SOA condition the picture is different, as there is no consistency effect for unrelated answers. We assume that unrelated probes can be rejected without full activation of the correct result based on familiarity judgments. As consistency of a probe is defined by its relationship to the correct result, there can be no consistency effect as long as no correct result is available. Yet, this is again different for related probes even for the short SOA: Related probes cannot be rejected without specific result-related knowledge, because they stem from the correct multiplication row. Therefore, it takes longer to reject related compared to unrelated probes. However, once the correct result has to be determined, the consistency of lure can come into play. This is exactly what we observed in the data.

### EEG-data

For both SOAs, we found a specific biphasic EEG pattern for lures – a negativity followed by a positivity. In analogy with previous ERP studies on visual lexical decision [26,30,31] and on the verification of simple multiplication problems [20,22,23], we interpret the negativity as an instance of the N400 effect, reflecting lexico-semantic processing, and the positivity as a late positive component (LPC), reflecting plausibility judgments and/or expectancy. Both effects were initiated earlier in the long than in the short SOA conditions due to the more advanced activation of the set of answer candidates (including the correct response) in the former compared to the latter at the onset of EEG recording.

A more detailed analysis of electrophysiological responses to different lure types revealed a distinctive influence of the factors relatedness and consistency. In the short SOA conditions, the factor relatedness played a role only in the late positive component between 450 and 650 ms, showing that unrelated probes were less plausible than related ones. No significant effect of decade-consistency was observed at this SOA. In contrast, in conditions with a long SOA the factor relatedness became evident in the N400 component as well as in the LPC. Unrelated lures produced an enhanced negativity compared to related lures in the time window between 300 ms and 400 ms and a more pronounced positivity effect between 475 ms and 600 ms after probe onset. Moreover, lures that are consistent with the correct result produced negativity effects of higher amplitude than inconsistent probes. With respect to the LPC component, we found a more pronounced positivity for inconsistent than for consistent lures.

Despite their occurrence within the same time window, the N400 modulation by the factor consistency differs from the one caused by the factor relatedness. As Jost, Niedeggen, and Rösler pointed out [20,22,23], related lures that are associatively connected with the correct solution produce *less *pronounced negativity effects in product verification than unrelated numbers. According to their interpretation, related lures are more strongly activated by the problem than unrelated probes. Our results seem to lend support to this interpretation.

In contrast to these findings, the present ERP data show that the negativity *increases *with formal similarity (i.e. consistency) between correct probes and lures, i.e. when the lure is a neighbor of the correct probe. Thus, the interpretation for relatedness proposed by Jost, Niedeggen, and Rösler [20,22,23] cannot explain our results for consistency. Rather, an explanation in terms of neighborhood frequency (lexical account') is favored by the data, similar to the one proposed for visual lexical decision [26,30,31]: A consistent lure which has formal overlap with a highly familiar answer to that problem (i.e. to the correct result) leads to a more pronounced negativity than an inconsistent lure which does not show such formal overlap with a familiar answer to that problem. Following this account, the enhanced N400 component for lures with familiar neighbors reflects a summation of semantic activation by the lure and by its lexical neighbor, i.e. the correct result [26,30,31]. Note that this lexical' interpretation does not necessarily exclude the existence of semantic effects caused by decade consistency. It may be that both semantic association and neighborhood consistency play a role, both affecting the EEG into different directions, but the latter dominating in the N400 time window. Interestingly, while semantic activation (e.g. the relatedness effect) and neighborhood (e.g. the consistency effect) modify the N400 in different directions, they have comparable behavioral consequences: Both relatedness and consistency lead to increased RTs and error rates.

The picture is different in the LPC time window. Here, the predictions of a semantic account [20] are fulfilled both for the relatedness and for the consistency manipulation: Unrelated as well as inconsistent probes lead to enhanced positivity compared to their related and consistent counterparts. However, it remains unclear why (lexical) neighborhood effects dominate in the N400 epoch, while semantic effects prevail in the LPC.

### The functional locus of the consistency effect

It is important to recall that the participants in our study had to give a dichotomous manual response. Therefore, formal similarities of consistent lures with the correct result in phonological or motor output (or its preparation) cannot explain our results. Hence, the consistency effect in the present study relies either on visual encoding or on lexico-semantic processing. In a previous study we have found evidence for consistency effects in the verbal production of solutions to simple multiplication problems [24]. Obviously, encoding for consistent and inconsistent errors does not differ in a production task, because only the multiplication problem is presented and the consistent or inconsistent errors are produced by the participants themselves. Therefore, differences in encoding cannot explain the consistency effect found in the production task. Rather, it must be based on lexico-semantic processes or response output preparation. If we take the results of both studies together, neither differences in encoding nor differences in response preparation or selection alone can explain both findings. However, differences in the lexico-semantic representation between consistent and inconsistent answers would explain both results in a uniform way. Therefore, we suggest that two-digit multiplication results are represented in a decomposed fashion at a lexico-semantic level of processing with separate representations of decades and units. This notion is in line with the IN model [5].

However, there is an alternative to a lexico-semantic locus of the consistency effect: One could presume that, on the one hand, differences in response selection cause the consistency effect in the production task and that, on the other, differences in encoding cause the consistency effect in the verification task. Thus, the effect would be observed in both tasks, however, for different – task specific – reasons. Our analyses of event-related potentials allow to disentangle both accounts. While the semantic account and the lexical account as outlined in the Background section differ in their specific predictions about the direction of EEG modulation in the N400 and late positive components, both accounts involve central lexico-semantic' processing rather than peripheral (i.e. encoding or response preparation) processes. Therefore, a central lexico-semantic' locus of the consistency effect is clearly supported by our ERP data. Note, however, that we do not exclude the possibility that consistency between correct result and lure additionally affects peripheral processing stages. Still, we want to highlight the conclusion that lexico-semantic representations of multiplication results seem to be decomposed into tens and units as predicted by the IN model [5].

This evidence for decomposed processing in multiplication converges with evidence for decomposed processing in other numerical tasks. Nuerk and colleagues [34-36] have shown in a series of behavioral and neuropsychological studies that the magnitude comparison of two-digit numbers is influenced by decomposed representations of tens and units. A similar finding was reported by Verguts and de Moor [37]. Furthermore, in a number bisection task, it has been shown that decade crossing (as an index of separate processing for units and decades) was one of the most important predictors in determining performance [38]. Finally, recent data in addition tasks suggest that the separate magnitudes of decades and units may be more important than the holistic magnitude of the problem [39]. We therefore suggest that whenever the semantic representations of multi-digit numbers are accessed – even when it is as automatic as in multiplication fact retrieval – decomposed representations of decades and units are activated, possibly in addition to a holistic representation of the multi-digit number.

## Conclusion

We have provided behavioral and electrophysiological evidence for neighborhood consistency effects in the verification of simple multiplication problems. Decade-consistent answers took longer to be rejected compared to inconsistent answers. In the EEG, consistent answers led to a more pronounced N400 component and inconsistent answers led to a more pronounced LPC effect. As predicted, the consistency effect appeared late compared to the relatedness effect, as more specific information about the correct result has to be activated for the former compared to the latter. To conclude, our results suggest that the consistency effect in simple multiplication is not only located at peripheral stages, but is indicative of the non-holistic organization of lexico-semantic knowledge for arithmetic facts.

## Abbreviations

ANOVA Analysis of Variance

EEG Electroencephalogram

EOG Electrooculogram

ERP Event-Related Potential

IN Interacting Neighbors (Model)

LPC Late Positive Component

N Negativity

P Positivity

RT Reaction Time

SOA Stimulus Onset Asynchrony

## Competing interests

The author(s) declare that they have no competing interests.

## Authors' contributions

FD, ER, TV, KW, and HCN conceived of the study. All authors participated in its design. UJ performed data collection and processing. FD, UJ, and HCN performed the statistical analyses. FD, UJ, TV, and HCN drafted the manuscript; the other authors revised it critically. All authors contributed to the interpretation of the data. All authors read and approved the final manuscript.

## Supplementary Material

Additional file 1 **Experimental stimuli**. This file provides an overview about the experimental stimuli used. Click here for file
