# Supplementary material for: Neighborhood consistency in mental arithmetic: Behavioral and ERP evidence
Source: Behav Brain Funct. 2007 Dec 28;3:66. doi: 10.1186/1744-9081-3-66 (PMC2265290; doi:10.1186/1744-9081-3-66)
Supplement: Additional file 1 — Experimental stimuli. This file provides an overview about the experimental stimuli used. [file 1744-9081-3-66-S1.pdf]

Problem	Operand 1	Operand 2	Correct result	Operand-related lures		Unrelated lures	
				consistent	inconsistent	consistent	inconsistent
3x6	3	6	18	12	**24**	13	**23**
6x3	6	3	18	15	**21**	17	**29**
4x5	4	5	20	**24**	16	**29**	17
5x4	5	4	20	**25**	15	**23**	13
3x7	3	7	21	**24**	18	**23**	19
7x3	7	3	21	**28**	14	**29**	17
5x5	5	5	25	20	**30**	23	**31**
4x7	4	7	28	24	**32**	26	**34**
7x4	7	4	28	21	**35**	23	**31**
5x6	5	6	30	**35**	25	**39**	29
6x5	6	5	30	**36**	24	**38**	26
8x4	8	4	32	**36**	28	**38**	26
7x5	7	5	35	30	**40**	31	**43**
5x8	5	8	40	**45**	35	**47**	39
8x5	8	5	40	**48**	32	**46**	38
7x6	7	6	42	**49**	35	**47**	31
8x6	8	6	48	42	**54**	41	**51**
7x7	7	7	49	42	**56**	46	**58**

Additional file 1: Experimental Stimuli. Probes larger than the correct result are printed in boldface.
